# Relationship between exposure to ionizing radiation and mesothelioma risk: A systematic review of the scientific literature and meta‐analysis

**DOI:** 10.1002/cam4.4436

**Published:** 2022-01-14

**Authors:** Giovanni Visci, Emanuele Rizzello, Carlotta Zunarelli, Francesco Saverio Violante, Paolo Boffetta

**Affiliations:** ^1^ IRCCS Azienda Ospedaliero‐Universitaria di Bologna Bologna Italy; ^2^ Public Health Department Local Health Unit of Imola Bologna Italy; ^3^ Department of Medical and Surgical Sciences University of Bologna Bologna Italy; ^4^ Stony Brook Cancer Center Stony Brook University Stony Brook New York USA

**Keywords:** ionizing radiation, mesothelioma, nuclear industry, radiotherapy

## Abstract

**Background:**

Ionizing radiation and mesothelioma have been examined among personnel employed in nuclear power plant and patients treated by external beam radiation therapy (EBRT). The association is still controversial; the purpose of this review is to summarize the scientific evidence published in the literature regarding the relationship between ionizing radiation and incidence of mesothelioma and, if possible, estimating strongness of the association by meta‐analysis of extracted data.

**Methods:**

Articles included in the systematic review were retrieved by searching among the three main scientific databases: PubMed, Scopus, and Embase. The literature search was conducted in June 2021. A meta‐analysis of random effects was conducted, stratified by exposure (EBRT, occupational exposure). The heterogeneity of the summary relative risks (RRs) was assessed using *I* ^2^ statistics. Publication bias was evaluated graphically through the funnel plot.

**Findings:**

The exposure to ionizing radiation could be a risk factor for mesothelioma: both for exposure to high doses for short periods (EBRT) (RR of 3.34 [95% confidence interval, CI 1.24–8.99]) and for exposure to low doses for a prolonged duration (exposure working) (RR of 3.57 [95% CI 2.16–5.89]).

**Conclusions:**

Despite the low number of mesotheliomas in the general population, the steadily increased risk among individuals exposed to radiation is still worth considering.

## Graphical abstract

The exposure to ionizing radiation could be a risk factor for mesothelioma: both for exposure to high doses for short periods (external beam radiation therapy) (relative risk [RR] of 3.34 [95% confidence interval, CI 1.24–8.99]) and for exposure to low doses for a prolonged duration (exposure working) (RR of 3.57 [95% CI 2.16–5.89]).

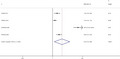

## 1 INTRODUCTION

Malignant mesothelioma is an infrequent neoplasm that usually arises from the lining cells of the pleural and peritoneal cavities. According to the evidence, asbestos is the most significant risk factor of mesothelioma^1^, ^2^; the cumulative lifetime risk of developing the disease in the absence of exposure to asbestos, has been estimated to be approximately 3 in 10,000.^3^ Recent epidemiological studies have focused on additional possible causal factors of mesothelioma, including asbestiform mineral fibers (erionite; fluoroadenine); carbon nanotubes; chronic serous inflammation; and ionizing radiation.^4^

Ionizing radiation is a well‐known human carcinogen.^5^ Mesothelia are considered to be not very radiosensitive tissues, however, some studies identified an increased risk of developing mesothelioma among subjects exposed to ionizing radiation.^4^

The link between ionizing radiation and mesothelioma has been investigated among nuclear power plant workers and through patients exposed to either Thorotrast (a contrast agent used for x‐rays) or external beam radiation therapy (EBRT). The available evidence on the association is still unclear for several reasons: most studies are based on a limited number of mesothelioma cases and there is a lack of consensus on the shape of the dose–response relationship for low doses.^6^

The purposes of this work are (1) to update the review of literature by Goodman et al.,^4^ trying to focus on both occupational and therapeutic exposure to radiation and excluding the diagnostic one; (2) to analyze state of evidence regarding the relationship between exposure to ionizing radiation and incidence of mesothelioma, carrying out a quantitative synthesis of the findings; and (3) to distinguish between the exposure to high doses for short periods (EBRT) and the exposure to low doses for a prolonged duration (exposure working).

The relevance of this study is notable and can be demonstrated by the two examples below.

Regarding therapeutic exposure, if it were conceivable a more than additive interaction between ionizing radiation and asbestos in determining the risk of mesothelioma, EBRT could become a second choice therapeutic option (favoring surgery) for the treatment of localized cancer among former workers with significant previous exposure to asbestos.

As regards occupational exposure, if a positive association between ionizing radiation and mesothelioma was to be demonstrated, in nuclear industries it would be necessary to implement protection and safety measures (minimize the doses emitted and better use of Personal Protective Equipment) and Health Surveillance measures (with more careful and frequent checks).

## 2 METHODS

### 2.1 Study selection

This systematic review was conducted according to the PRISMA statement.^7^ The study protocol was registered with PROSPERO at https://www.crd.york.ac.uk/prospero/ (registration number: CRD42021259581).

Articles included were identified using PubMed, Scopus, and Embase databases. The literature search was conducted in June 2021.

The following string was created, using PubMed and then adapted for Scopus and Embase (see in the Appendix 1):

(Radiotherapy OR EBRT OR “external beam radiotherapy” OR “stereotactic radiotherapy” OR (peritoneal AND irradiation) OR radionuclides OR “therapeutic ionizing radiation”) OR ((Nuclear AND (industry * OR work OR worker * OR job)) OR (Radiation AND (industry * OR work OR worker * OR job)) OR “Radiography / *adverse effects” [Mesh] OR (hiroshima [tiab] OR (nagasaki [tiab] OR atomic bomb survivors OR life span study) OR “Thorium Dioxide [Mesh] OR Thorotrast”*.

AND

(“Etiology” [Subheading] OR etiologic * OR Neoplasm, *Radiation*‐*Induced [MH] OR Neoplasms*, *Radiation*‐Induced * OR Neoplasms, *Second Primary [MH] OR Neoplasms*, Second Primary * OR etiology [MH] OR aetiologic * OR aetiology OR Cohort Studies [MH]))).

AND

*“Mesothelioma” OR “pleural cancer / neoplasm” OR “peritoneal cancer / neoplasm”*.

Empirical validity of the search strategy was considered good as none of the articles relevant, already owned, was not unretrieved by the reported string.

### 2.2 Inclusion criteria

The review included articles that met the following criteria: cohort or case–control design, information about exposure to ionizing radiation, and mesothelioma as outcome. No restrictions were applied either for the year of publication or for the language of publication.

### 2.3 Selection process

**FIGURE 1:**
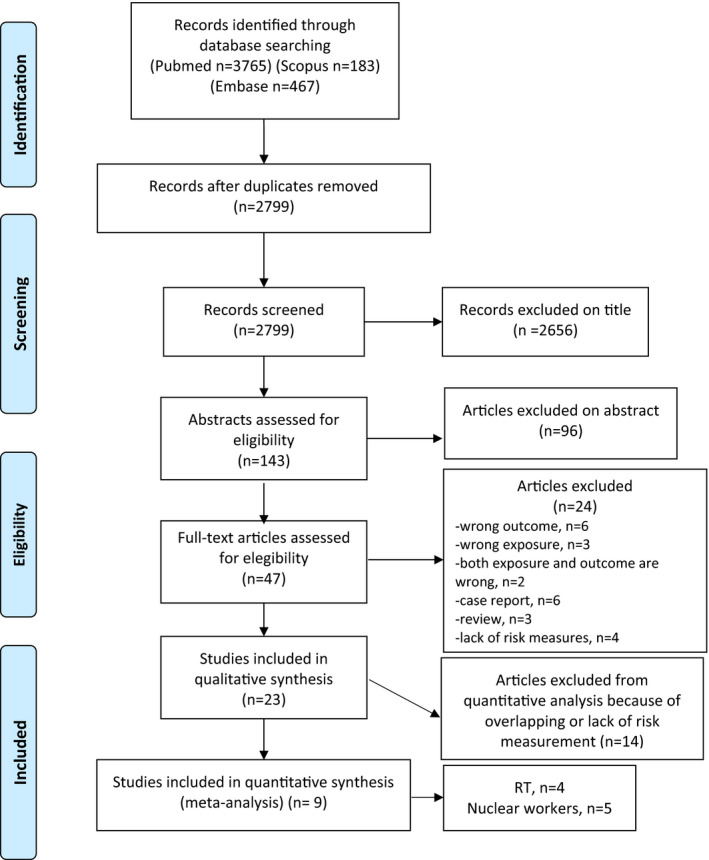
Flowchart for the identification of articles for the meta‐analyses (PRISMA)

Two authors (ER and GV) independently performed the search using the aforementioned strings, examined the lists of titles and abstracts to exclude irrelevant and duplicate articles. In case of disagreement (3.57 [95% confidence interval, CI 2.16–5.89]), a third reviewer (CZ) was consulted. Subsequently, the full texts of the potentially relevant articles were independently analyzed by the two authors (ER and GV) and the studies that met the inclusion criteria were identified. The references listed in the articles retained as relevant were reviewed to identify additional studies. The flow chart of the selection process is shown in Figure 1.

### 2.4 Data extraction

The following information was extracted from the text by two authors (ER and GV): year of publication, country, study design, age and gender distribution of the study population, study period, loss to follow‐up or rate of response (for case–control studies), cohort size or number of cases and controls, source of data on exposure and outcome, type of outcome (mortality or incidence), measure of association (relative risk [RR] or standardized mortality ratio), and corresponding CI. In a preliminary step, the stratified results were combined in a single risk estimate. Data extraction was performed by two authors (ER and GV) and was checked by a third author (CZ).

### 2.5 Quality assessment

A checklist for the evaluation of the quality of the studies was used, drawn up on the basis of the National Institutes of Health (NIH) indications and adapted for a better evaluation of the articles subject to the systematic review.^8^ In particular, the following aspects were taken into consideration: the presence of known exposure to asbestos, a follow‐up period of over 10 years, and average age of the study population above 60 years.

### 2.6 Statistical analysis

A meta‐analysis was conducted^9^ using the Stata 14 package (StataCorp), using specific commands metan, metabias, and metafunnel. We performed an analysis stratified by exposure (EBRT, occupational exposure). The heterogeneity of the synthetic RRs was assessed using *I* ^2^ statistics.^10^ We considered the funnel plot and performed the Egger's regression asymmetry test to assess publication bias.

## 3 RESULTS

The search identified 4415 articles (3765 in PubMed, 183 in Scopus, and 467 in Embase); duplicates were excluded. Of the remaining 2799 potentially relevant studies, 2656 were excluded on the basis of the title and 96 on the basis of the abstract.

A total of 47 articles were evaluated against the inclusion criteria, and the review of the full text led to the exclusion of 23 of them. Additionally, 14 papers were not included in quantitative synthesis because of overlaps or lack of information concerning exposure measurement.

Finally, nine articles were included in the quantitative analysis (Tables 1 and 2). Risk estimates for mesothelioma are shown in Tables 3 and 4. All the articles included are cohort studies, four concern exposure to post‐neoplastic radiotherapy, and five investigate occupational exposure of nuclear power plant workers (no studies of Thorotrast patients were retained in the review).

**TABLE 1:** Characteristics of radiotherapy studies

Reference	Country	Sex (%M)	Study period	Cohort size	Person years	Source of data on exposure	Exposure	Source of data on outcome	Outcome	Age	Quality score	Studies with overlapping cohorts
Chang et al.^11^	USA	54	1973–2014	299,309	2,010,600	SEER 18	RT	SEER 18	Incidence	NA	61	Teta et al.^12^
Farioli et al.^13^	USA	33	1973–2012	935,637	NA	NA	RT	NA	Incidence	59.7	78	Farioli et al.^13^, Berrington de Gonzalez et al.^14^, Neugut et al.^15^
De Bruin et al.^16^	Netherlands	56	1965–1995	2567	46462.7	Five cancer centers/University hospitals in Netherlands	RT	Five cancer centers/University hospitals in Netherlands	Incidence	NA	83	/
Pickles et al.^17^	Canada	100	1984–2000	39,261	142,983	BC Cancer Registry	RT	BC Cancer Registry	Incidence	71.5	61	/

Abbreviations: NA, not available; RT, radiotherapy; SEER, Surveillance, Epidemiology, and End Results.

**TABLE 2:** Characteristics of nuclear workers studies

Reference	Country	Sex (%M)	Study period	Cohort size	Person years	Source of data on exposure	Exposure	Source of data on outcome	Outcome	Age	Quality score	Overlapping studies
Samson et al.^18^	France	87.9	1968–2008	12,649	342,258	TRACY	Uranium	CépiDC–INSERM and National Vital Status registry	Mortality	NA	51.1	Metz‐Flamant et al.^19^, Telle‐Lamberton et al.^20^
Schubauer Berigan et al.^21^	USA	80.4	Up to 2015	119,196	4,019,065	Hanford, INL, ORNL, PNS, and SRS	Ƴ ray, neutrons, and tritium	National Death Index	Mortality	NA	55.6	/
Matanoski et al.^22^	USA	100	1957–1982	71,815	920,907	US Shipyard Workers	Nuclear industry	Social security administration files, civil service administration files, HCFA files, Virginia mortality files, and National Death Index	Mortality	NA	61.1	/
Habib et al.^23^	Australia	74.2	1972–1998	7023	128036.1	LHSTC	Nuclear industry	INSERM	Mortality	NA	55.6	Habib et al.^24^
Carpenter et al.^25^	United Kingdom	100	1946–1988	75,006	NA	AEA establishment at Harwell, AWE establishment at Harwell, AWE, and Sellafield	Plutonium, tritium, and other radionuclides	NHSCRs	Mortality	NA	45.6	Omar et al.^26^, Atkinson et al.^27^

Abbreviations: HCFA, Health Care Financing Administration files; INL, Idaho National Laboratory; INSERM, French National Health and Medical Research Institute; LHSTC, Lucas Heights Science and Technology Centre; NA, not available; NHSCRs, National Health service central registers; ORNL, Oak Ridge National Laboratory; PNS, Portsmouth Naval Shipyard; SRS, Savannah River Site; TRACY, Travailleurs du cycle.

**TABLE 3:** Risk estimates for mesothelioma in radiotherapy studies

Reference	Country	Person years	Number of mesothelioma	RR/SIR	Index	LbCI	UbCI	Note
Chang et al.^11^ ^,^ ^a^	USA	2,010,600	28	RR	1.64	1.05	2.57	/
Chang et al.^11^ ^,^ ^a^	USA	2,010,600	28	SIR	1.78	1.18	2.58	/
Farioli et al.^13^	USA	NA	301	RR	1.34	1.04	1.74	/
De Bruin et al.^16^	Netherlands	46462.7	13	SIR	25.7	13.7	44	/
Pickles et al.^17^	Canada	142,983	28	SIR	2.28	1.55	3.25	*p* = 0.003
Pickles et al.^17^	Canada	142,983	28	SIR	2.55	NA	NA	<5 years
Pickles et al.^17^	Canada	142,983	28	SIR	1.55	NA	NA	5–10 years
Pickles et al.^17^	Canada	142,983	28	SIR	3.06	NA	NA	>10 years

Abbreviations: LbCI, lower bounder confidence interval; NA, not available; RR, relative risk; SIR, standardized incidence ratio; UbCI, upper bounder confidence interval.

^a^ The risk estimate was calculated for the studies that had multiple estimates.

**TABLE 4:** Risk estimates for mesothelioma in nuclear workers studies

Reference	Country	Person years	Number of mesothelioma	RR/IRR/SIR	Index	LbCI	UbCI	Note
Samson et al.^18^	France	342,258	17	SMR	2.04	1.19	3.27	NA
Schubauer Berigan et al.^21^	USA	4,019,065	96	SMR	2.8	2.27	3.42	NA
Matanoski et al.^22^ ^,^ ^a^	USA	920,907	36	SMR	5.11	3.03	8.08	>5 mSv
Matanoski et al.^22^ ^,^ ^a^	USA	920,907	36	SMR	5.75	2.48	11.33	<5 mSv
Habib et al.^23^	Australia	128036.1	5	SMR	21.11	8.79	50.72	NA
Carpenter et al.^25^ ^,^ ^a^	United Kingdom	NA	1	RR	0.48	0.03	2.69	Tritium
Carpenter et al.^25^	United Kingdom	NA	1	SMR	1.15	NA	NA	Tritium
Carpenter et al.^18^ ^,^ ^a^	United Kingdom	NA	9	RR	1.97	0.71	5.49	Plutonium
Carpenter et al.^25^ ^,^ ^a^	United Kingdom	NA	9	SMR	3.57	NA	NA	Plutonium
Carpenter et al.^25^ ^,^ ^a^	United Kingdom	NA	4	RR	1.62	0.38	6.27	Other radionuclides
Carpenter et al.^25^ ^,^ ^a^	United Kingdom	NA	4	SMR	2	NA	NA	Other radionuclides

Abbreviations: LbCI, lower bounder confidence interval; NA, not available; RR, relative risk; SIR, standardized incidence ratio; SMR, standardized mortality ratio; UbCI, upper bounder confidence interval.

^a^ The risk estimate was calculated for the studies that had multiple estimates.

**FIGURE 2:**
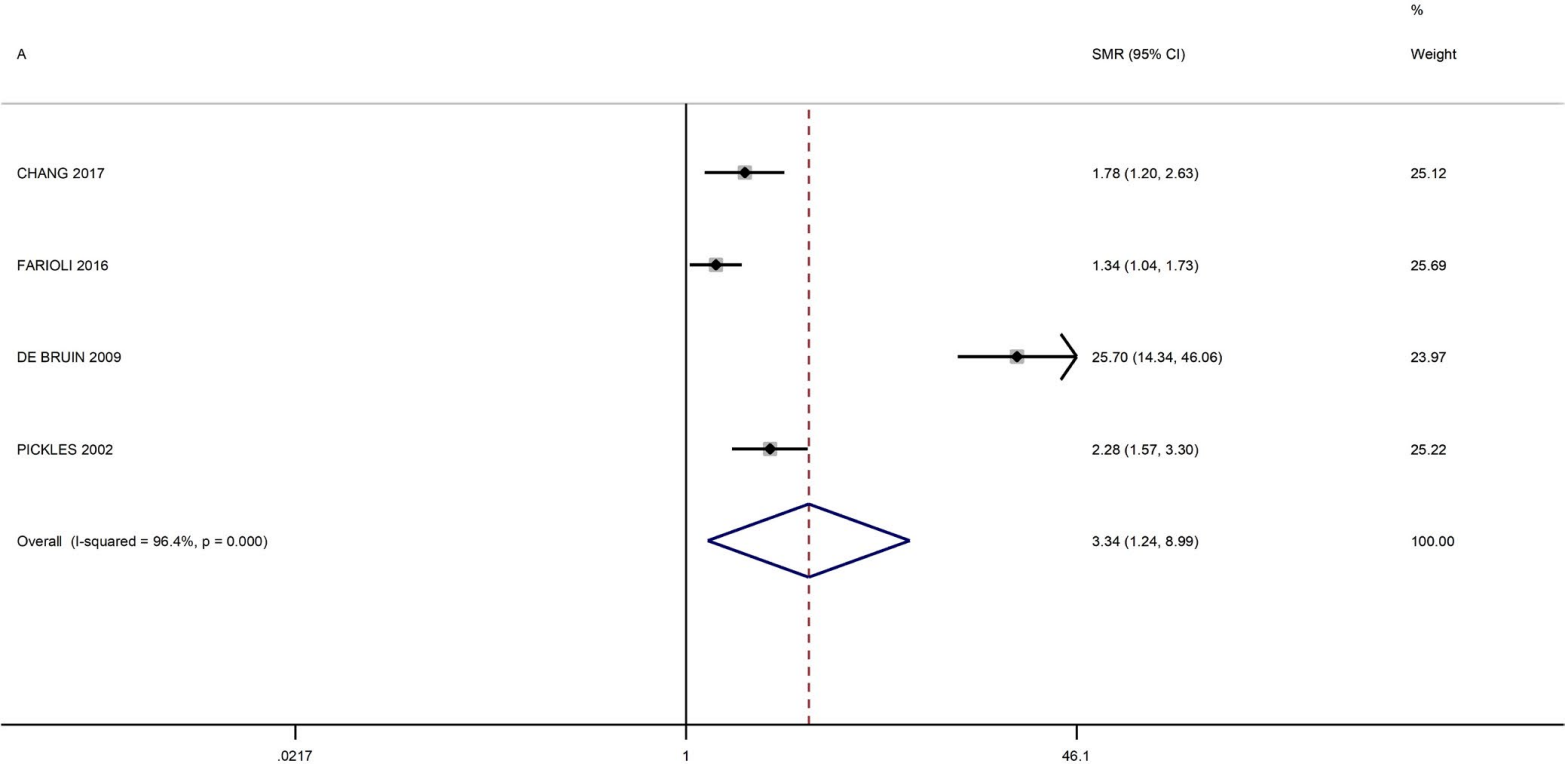
Forest plot of meta‐analysis of radiotherapy studies

**FIGURE 3:**
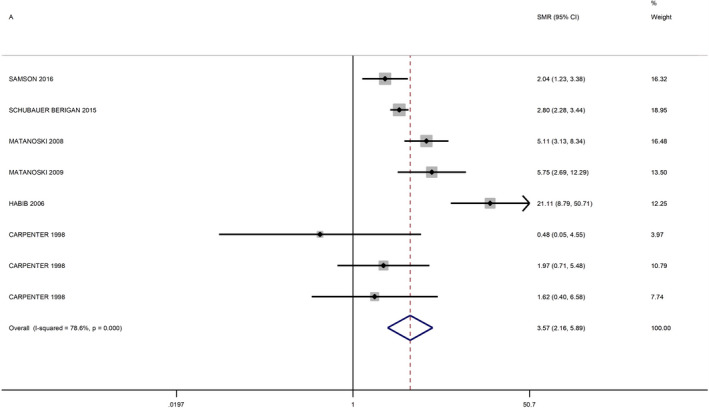
Forest plot of meta‐analysis of radiotherapy studies (excluding the study by De Bruin et al.^16^)

**FIGURE 4:**
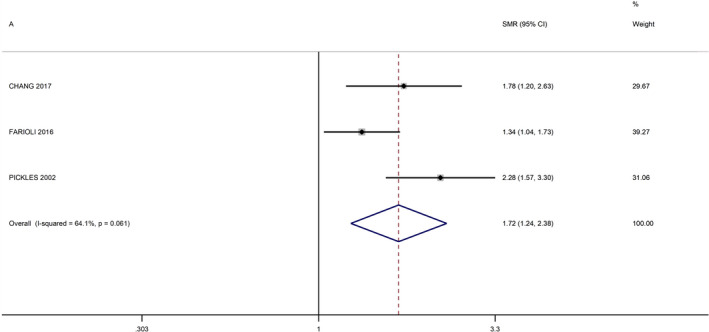
Forest plot of meta‐analysis of nuclear workers studies

No additional studies were identified from the review of the references lists. The meta‐analysis of studies among EBRT patients, resulted in a summary RR of 3.34 (95% CI 1.24–8.99) (Figure 2). The study by De Bruin et al.^16^ appeared to be an outlier, and its exclusion reduced the heterogeneity of the meta‐analysis from 96.4% to 64.1% (Figure 3). After this exclusion, the summary RR was 1.72 (95% CI 1.24–2.38). The meta‐RR of the studies among nuclear workers was 3.57 (95% CI 2.16–5.89) (Figure 4).

We did not analyze data about the association between Thorotrast and mesothelioma, because we decided to exclude them in order to reduce heterogeneity of exposure to ionizing radiation.

Patients treated with Thorotrast are exposed to alpha particles, while radiotherapy and workers employed in nuclear industry are exposed mainly to external radiation.

Furthermore, only few papers considered co‐exposure to asbestos as a confounding factor. In fact, in all the studies reviewed, asbestos exposure was cited as a possible confounder factor but none of the articles reported a precise estimation of the extent of exposure.

**FIGURE 5:**
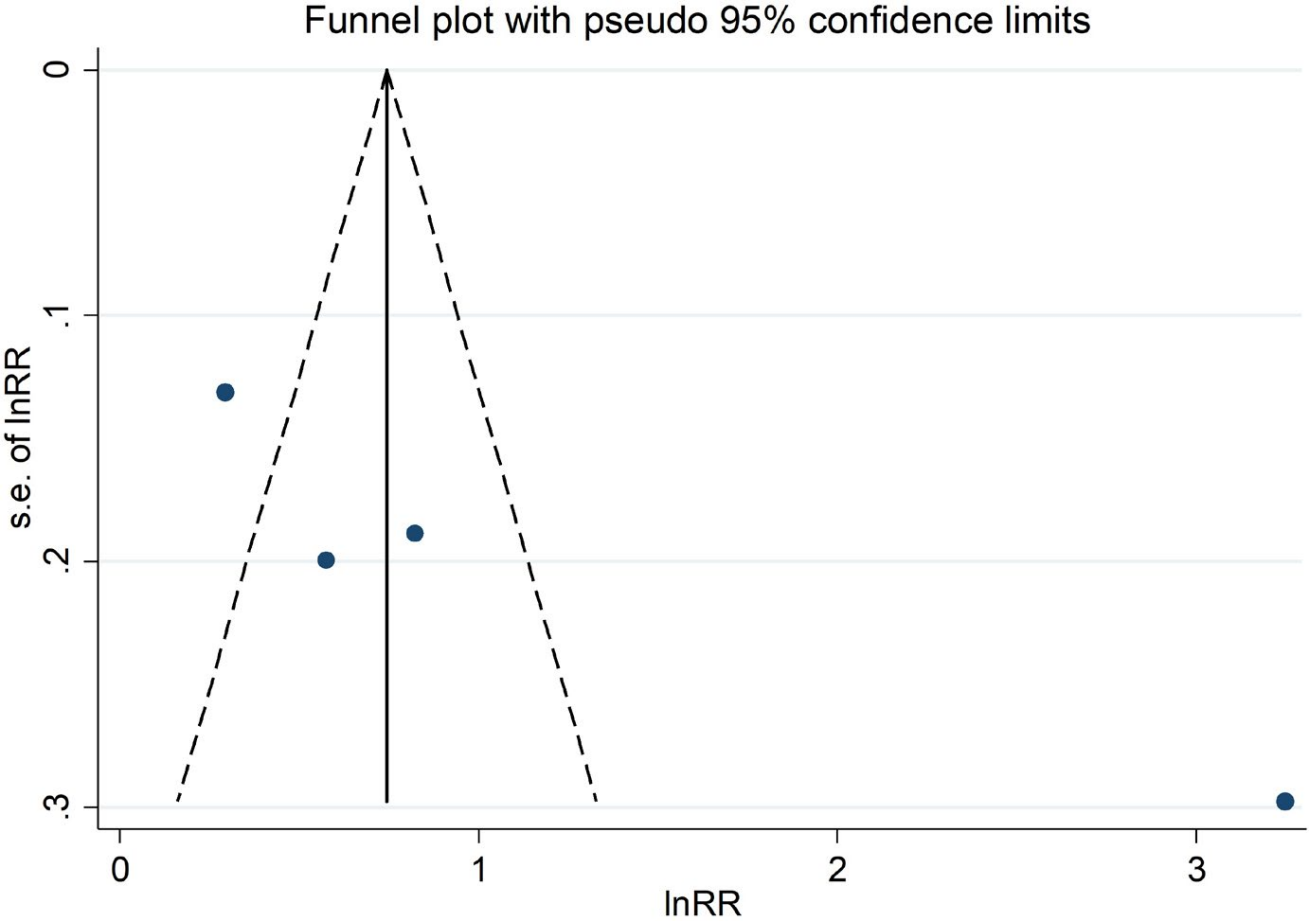
Funnel plot of the Egger's test to assess publication bias among radiotherapy studies

**FIGURE 6:**
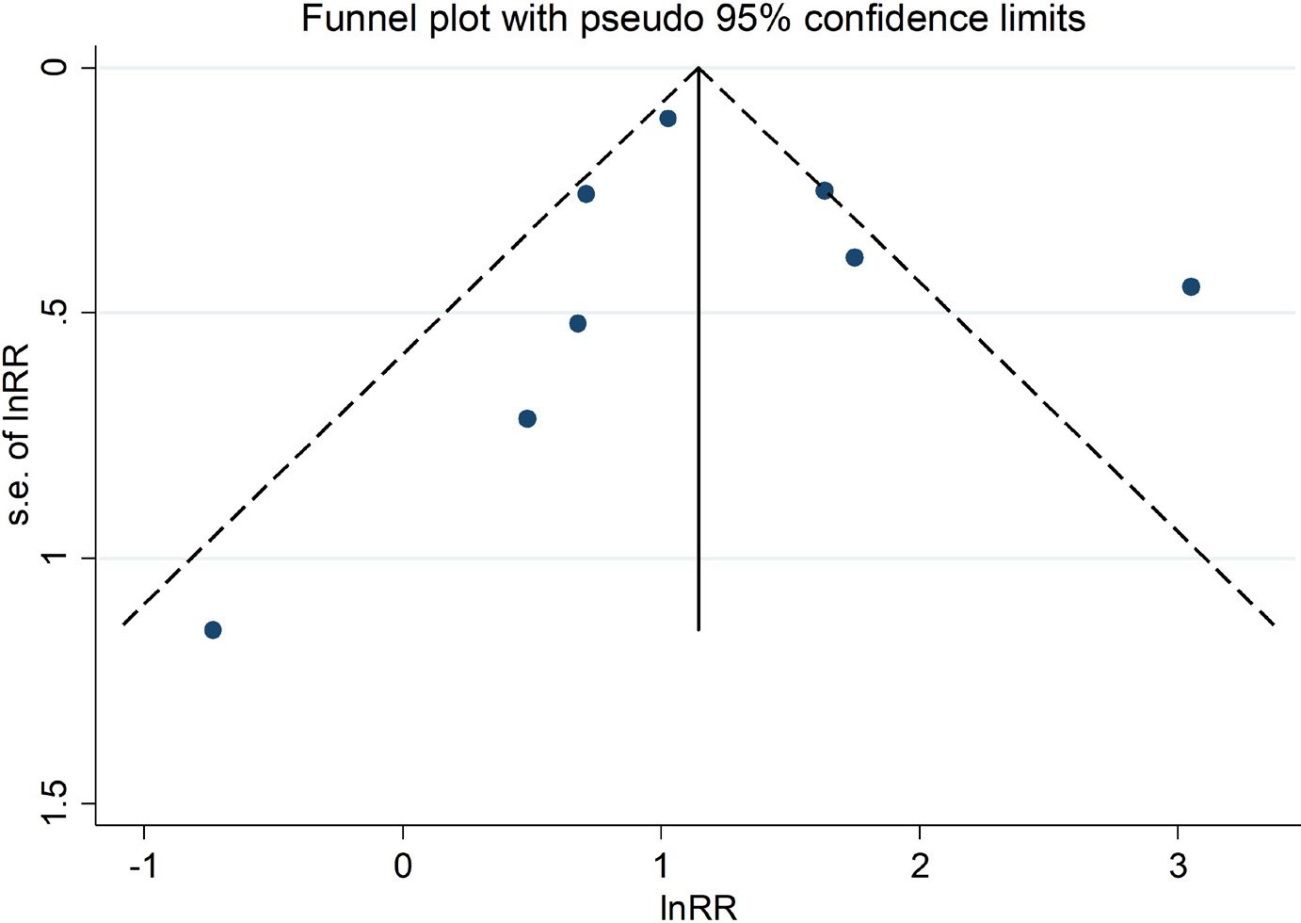
Funnel plot of the Egger's test to assess publication bias among nuclear workers studies

Finally, we found no evidence of publication bias in the main results of the cohorts included in the meta‐analysis, both for radiotherapy patients and nuclear workers (Figures 5 and 6).

## 4 DISCUSSION

Our analysis supports the hypothesis that exposure to ionizing radiation can lead to a significant increased risk of developing mesothelioma. Over the years have been reported many cases that have highlighted this association, especially for those cases in which patients develop a cancer of the mesothelium after radiotherapy treatments for primary tumors in other locations.^28^, ^29^ When the primary tumor is located in the abdominal‐pelvic area, given the closer proximity to the irradiation site, the association is stronger for peritoneal mesothelioma, likewise, for pathologies such as radiotreated Hodgkin's or non‐Hodgkin's lymphomas there is the possibility of developing pleural mesotheliomas.^13^ We could not evaluate this aspect because of lack of information: many authors did not consider pleural and peritoneal cancer separately and most of articles, included in quantitative analysis, did not show results about it.

An association between Thorotrast and mesothelioma has been previously reported^30^, ^31^; in this review, no studies on this association were analyzed since those articles found did not fall within the inclusion criteria.

As evidenced by Goodman et al.,^4^ the rarity of mesothelioma and its possible diagnostic misclassification represent important hurdles for epidemiological studies.

The main limitation of this research is in fact represented by the mesothelioma misclassification within the various studies, as can be seen from the search string both the words “mesothelioma” and “pleural cancer / neoplasm” or “peritoneal were used cancer / neoplasm”.

The lack of standardization in the reporting of malignant mesothelioma is a primary source of misclassification of the disease state. Pleural mesothelioma was not identified as a distinct cancer until 1960 and peritoneal mesothelioma was not identified until 1964 (possibly when the Surgeon General's report on smoking^32^ in 1964 boosted public awareness about the link between smoking and lung cancer).^33^

Mesotheliomas could have been diagnosed as abdominal cancer or pleural metastatic adenocarcinoma. This resulted in significant disease misclassification, which was not corrected in many cases until death records or autopsy files were carefully checked. This may lead to an underestimation of the number of mesotheliomas diagnosed affecting the final result of the meta‐analysis.

The studies included in our meta‐analysis that refer to occupational exposure detect the disease state mostly from National Death Registers, in which, as explained above, the diagnosis is not always reported adequately; furthermore, immunophenotypic patterns are not always specified.

In studies related to therapeutic exposure, however, the outcome usually comes from the SEER registries or from National Cancer Registries (e.g., BC Cancer Registry); in this context, the quality of the diagnosis is certainly higher, but biases from misclassification cannot be excluded.

Lack of information concerning co‐exposure to asbestos did not allow to correct the estimate for this variable. In all the studies examined, asbestos exposure was cited as a possible confounder, but none had a precise estimate of the extent of exposure. Furthermore, studies conducted among occupational cohorts do not have information on dose and time elapsed from the exposure. For the reasons mentioned above, the quantification of the absolute risk of mesothelioma and the possible interaction between exposure to ionizing radiation and asbestos in determining the risk of malignant mesothelioma could be biased. The little information available to us about a precise and timely estimate of exposure to ionizing radiation (both on external irradiation and on contamination) did not allow us to study the dose–response relationship.

As regards the type of exposure to ionizing radiation, we know that for patients exposed to radiotherapy, the sources are represented by radiation that has similar linear energy transfer (x‐ray and Ƴ‐ray). Likewise in nuclear industry, the source of exposure is very variable and not always with homogeneous characteristics (uranium, plutonium, tritium, and other radionuclides); not many studies bring data on the different types of radiation and this inevitably turns out to be a source of uncontrolled variability.

In a single conventional radiotherapy session, the absorbed dose typically ranges from 1500 to 2000 mSv. However, typically, these doses are divided into multiple smaller doses which are given up over a period of 1–2 months. The specific dose for each patient depends on the location and severity of the tumor. Dose determination is therefore at the discretion of the radiation oncologist who is responsible for these treatment decisions.

For occupational exposure the effective (cumulative) dose limit is 20 mSv per year; it is therefore an enormously lower amount of radiation than the therapeutic one.

Therefore, there does not appear to be a dose–response gradient in the results of our meta‐analysis.

Another problem is that the meta‐analysis can examine events that were too rare in the original studies to show a statistically significant difference. However, the analysis of rare events is a problem because changes in the data can determine large changes in the results and this instability can be exaggerated by relative measures of effect instead of absolute.^34^ In the studies included in this work it is not always ascertainable the absolute risk value and therefore the reference level; for this reason, the final result will be affected by bias as there is an additional source of unmeasured variability.

In reality, rather than reporting a synthetic impact, the purpose of a meta‐analysis should be to synthesize effect sizes. If the effects are consistent, the analysis reveals that they are robust across all trials. If there is a small amount of dispersion, it serves to contextualize the average effect. If there is a significant dispersion, the focus should shift away from the synthetic effect and toward the dispersion itself. Researchers who report a synthesis and heterogeneity effect are missing the crucial synthesis.^35^

The results of our meta‐analysis confirmed that a relationship between exposure to ionizing radiation and the incidence of mesothelioma could be assessed. In addition, differences in the distribution of data were highlighted between the studies with radiotherapy exposure (North America for the most part) and those studying occupational exposures (United Kingdom, France, and Australia).

Several studies conducted among survivors of atomic bombs, and other nuclear disasters, did not explore the carcinogenic effect for small and prolonged doses over the time,^36^, ^37^ on the other hand, we try to examine that aspect.

It must be considered that since malignant mesothelioma is a rare cancer, the number of cases examined, considering the number of studies and the number of subjects, is always low. This does not allow to have a high statistical power.

A strength of our analysis is the consistency of results across different subgroups of studies included in the meta‐analysis. Furthermore, the limited heterogeneity between study results and the lack of evidence of publication bias, support the main conclusion of our meta‐analysis.

## 5 CONCLUSIONS

Our review and meta‐analysis show that exposure to ionizing radiation could be a risk factor for mesothelioma: both for exposure to high doses for short periods (EBRT) and for exposure to low doses for a prolonged duration (exposure working). Despite the low number of mesotheliomas in the general population, the steadily increased risk among individuals exposed to radiation is still worth considering.

More detailed studies on asbestos‐ionizing radiation co‐exposure would be needed and should be conducted to understand the role of both in mesothelioma development.

To this, it should be added more detail on the coding of mesothelioma, using a precise (and currently available) classification of the disease.

Considering the estimates obtained, the clinical cases and the reasonable type of action, evidences support a causal connection between exposure to ionizing radiation and the risk of mesothelioma, but we are unable to understand the role of the other confounding elements (asbestos, other carcinogens, etc).

In future studies, further investigations should increase the accuracy and detail of asbestos exposure and provide a consistent measure of exposure to ionizing radiation, considering the risk of malignant mesothelioma.

## DECLARATIONS

All methods were carried out in accordance with relevant guidelines and regulations. As this review and meta‐analysis were a retrospective observational study, no questions were asked to the ethics committee.

## CONFLICT OF INTEREST

The authors declare no conflict of interest.

## Data Availability

All the primary data are available from the first author.

## APPENDIX 1

### RESEARCH STRINGS

1) PubMed

((((Radiotherapy OR EBRT OR “external beam radiotherapy” OR “stereotactic radiotherapy” OR (peritoneal AND irradiation) OR radionuclides OR “therapeutic ionizing radiation”)) OR ((Nuclear AND (industry* OR work OR worker* OR job)) OR (Radiation AND (industry* OR work OR worker* OR job))))) OR (“Radiography/adverse effects”[Mesh]) OR (hiroshima[tiab] OR nagasaki[tiab] OR atomic bomb survivors OR life span study) OR ("Thorium Dioxide"[Mesh] OR Thorotrast)

AND

(“etiology”[Subheading] OR etiologic* OR Neoplasm, Radiation‐Induced[MH] OR Neoplasms, Radiation‐Induced* OR Neoplasms, Second Primary[MH] OR Neoplasms, Second Primary* OR etiology[MH] OR aetiologic* OR aetiology OR Cohort Studies[MH])

AND

(Mesothelioma OR Pleural cancer OR Pleural neoplasm OR Peritoneal cancer OR Peritoneal neoplasm OR “Mesothelioma”[MH] OR Mesothelioma/etiology* OR Pleural Neoplasms/mortality OR(Asbestos* AND (Cancer AND Neoplasm)))

2) Scopus

((TITLE‐ABS‐KEY (industry* OR work OR worker* OR job)) AND (TITLE‐ABS‐KEY (radiation))) OR ((nuclear) AND (TITLE‐ABS‐KEY (industry* OR work OR worker* OR job))) OR (radiotherapy OR ebrt OR “external beam radiotherapy” OR “stereotactic radiotherapy”) OR (radionuclides OR “therapeutic ionizing radiation”) OR (peritoneal AND irradiation) OR ((Radiography AND adverse effects) OR ((hiroshima OR nagasaki OR (atomic AND bomb AND survivors) OR (life AND span AND study)) OR ((Thorium AND Dioxide) OR Thorotrast))

AND

etiolog* OR aetiolog* OR (neoplasm AND radiation‐induced) OR (neoplasms AND second) OR (neoplasm AND primary)

AND

((cancer OR neoplasm) AND (asbestos)) OR (mesothelioma OR pleural AND cancer OR pleural AND neoplasm OR peritoneal AND cancer OR peritoneal AND neoplasm OR “Mesothelioma” [mh] OR mesothelioma/etiology* OR pleural AND neoplasms/mortality)

3) Embase

1) exp radiotherapy/ or exp external beam radiotherapy/ or exp adjuvant radiotherapy/ or exp “patient history of radiotherapy”/ or exp cancer radiotherapy/
2) exp radioisotope/
3) exp radiation exposure/ or exp ionizing radiation/
4) exp nuclear industry/
5) exp nuclear energy/
6) exp “Radiography adverse effects”/
7) exp hiroshima/ or exp nagasaki/ or exp atomic bomb survivors/ or exp life span study
8) exp Thorium Dioxide/ or exp Thorotrast
9) exp radiation carcinogenesis/ or exp second cancer/ or exp radiation‐induced neoplasm/
10) exp etiology/
11) exp pleura mesothelioma/ or exp mesothelioma/ or exp peritoneum mesothelioma/
12) 1 or 2 or 3 or 4 or 5 or 6 or 7 or 8
13) 9 or 10
14) 11 and 12 and 13
